# Plasma fatty acid profile is related to cognitive function in obese Chinese populations (35–64 years): A cross‐sectional study

**DOI:** 10.1002/fsn3.1738

**Published:** 2020-08-07

**Authors:** Qi Duan, Rong Fan, Ruqing Lei, Weiwei Ma, Bingjie Ding

**Affiliations:** ^1^ Department of Endocrinology Beijing Friendship Hospital Capital Medical University Beijing China; ^2^ School of Public Health Beijing Key Laboratory of Environmental Toxicology Capital Medical University Beijing China; ^3^ Department of Clinical Nutrition Beijing Friendship Hospital Capital Medical University Beijing China

**Keywords:** cognitive function, fatty acids, obesity, overweight

## Abstract

**Background:**

A fast‐growing body of evidence suggests that dietary lipids influence cognition, but the effects of dietary fatty acid (FA) intake and plasma FA profile on cognitive function in obese populations are currently unclear. The present study aimed to investigate the dietary FA intakes and plasma FA composition and their association with cognitive functions in obese and overweight populations aged 35–64 years.

**Methods:**

A total of 672 subjects were recruited and divided into normal‐weight, overweight, and obese groups based on their body mass index (BMI). Dietary information was collected using a semiquantified food frequency questionnaire. Plasma FAs composition was examined using gas chromatography. The mini‐mental state examination and Montreal Cognitive Assessment scales were carried out to assess the cognitive performance of each participant. Dietary FA intake and plasma FA composition were compared with rank transformation followed by one‐way ANOVA analysis across different BMI groups. Spearman rank correlation analysis was used to investigate the correlation between dietary FA intake and plasma FA composition and cognitive functions in normal‐weight, overweight, and obese subjects, respectively.

**Results:**

Overweight and obese subjects consumed larger amounts of saturated fatty acids (SFAs) compared to normal‐weight participants (*p < *.05). Obese populations also had higher plasma levels of total SFAs and total monounsaturated fatty acid (MUFAs) than normal‐weight subjects (both *p < *.05). In addition, plasma levels of SFAs, polyunsaturated fatty acids (PUFAs), and MUFAs were negatively correlated with cognitive functions in obese subjects but showed no correlation in normal‐weight and overweight subjects.

**Conclusions:**

From current data, we found higher plasma levels of SFA, PUFA, and MUFA in obese populations, which were associated with declined cognition. Lowering plasma FA levels may help maintaining normal cognitive functions in obese people.

## Graphical abstract

The present study aimed to investigate the dietary fatty acids (FA) intakes and plasma FA composition and their association with cognitive functions in obese and overweight populations. From current data, we found higher plasma levels of saturated fatty acid, polyunsaturated fatty acid, and monounsaturated fatty acid were associated with declined cognition of obese populations.

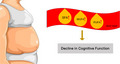

## 1 INTRODUCTION

Being overweight or obese is a key risk factor for a variety of chronic disorders, including cardiovascular disease, diabetes, dyslipidemia (Anari, Amani, Latifi, Veissi, & Shahbazian, 2017; Mathieu, Lemieux, & Despres, 2010; Sokolova et al., 2017) and neurodegenerative diseases (Ashrafian, Harling, Darzi, & Athanasiou, 2013; Pugazhenthi, Qin, & Reddy, 2017). Recent studies have shown that across the whole spectrum of lifespan, cognitive performance is significantly lower in obese populations than in people with normal weight, and it is negatively correlated with body mass index (BMI; Benito‐Leon, Mitchell, Hernandez‐Gallego, & Bermejo‐Pareja, 2013; Kim, Kim, & Park, 2016; Suemoto, Gilsanz, Mayeda, & Glymour, 2015). Obesity is generally associated with cognitive impairments independent of other socioeconomic or health‐related factors (Beilharz, Maniam, & Morris, 2015). Therefore, obesity represents a critical risk factor for cognitive dysfunction.

An unbalanced diet rich in fat has been widely recognized as a risk factor for obesity (Lee et al., 2011), and the composition of dietary fat and fatty acid (FA) also played a key role in obesity (Guerendiain et al., 2018). Previous prospective studies have shown variable associations of dietary intake of saturated fatty acids (SFAs), monounsaturated fatty acids (MUFAs), and trans‐unsaturated fatty acids (TFAs) with cognitive decline. For example, a high intake of SFA has been shown to contribute to obesity‐related disorders (Silva et al., 2017). Cross‐sectional and longitudinal studies showed that SFA intake was associated with global cognitive function, impairments in prospective memory, memory speed and flexibility, and neurological diseases (Eskelinen et al., 2008; Okereke et al., 2012). In contrast, higher intakes of PUFA and higher PUFA to SFA ratio were associated with better mobility and cognition in both children and elders (Gispert‐Llaurado et al., 2016; Jyvakorpi et al., 2017). These studies indicate that the quality and type of fat may have more impact on cognition than total fat intake, but this is difficult to ascertain in human populations. Therefore, the role(s) of varied dietary FA intake in obesity‐related cognitive dysfunction is still largely unknown.

In obese people, the composition of blood and tissue FAs is altered, and this affects important physiological functions in fat metabolism. Several studies have shown that obese adolescents had higher plasma levels of SFAs and lower plasma levels of MUFAs, docosahexaenoic acid (C22:6*n*−3, DHA) and total *n*−3 PUFAs than normal‐weight counterparts (Klein‐Platat, Drai, Oujaa, Schlienger, & Simon, 2005; Steffen et al., 2008), and these differences may contribute to cognitive dysfunction in obese people. However, there is a paucity of basic research of the association between FAs intake and the decline in the individual components of cognitive function rather than the total score of the standard mini‐mental state examination (MMSE) and Montreal Cognitive Assessment (MoCA) Scale in the obese population. Although there is solid evidence from patients with depressive episodes (Patra, Khandelwal, Chadda, Lakshmy, & Abraham, 2018) and from children with autism(Jory, 2016), supporting the idea that plasma FA composition is critical for neurodevelopment, there remains poor understanding of the association between diet fats and altered plasma FA profile and cognitive function in obese populations.

In the present study, we firstly compared the dietary FA intake and plasma FA composition among obese, overweight, and normal‐weight population (35–64 years). We then further analyzed the relationships between diet FAs, plasma FA profile, and cognitive performance in obese subjects.

## 2 MATERIALS AND METHODS

### 2.1 Study population

We recruited 672 subjects (305 males and 367 females) from the Daxing District of Beijing, aged 35–64 years. These subjects were classified into normal‐weight (*n* = 197), overweight (*n* = 260) and obese (*n* = 215) groups based on their BMI according to Chinese obesity criteria: a BMI between 18.5 and 23.9 is defined as normal weight; a BMI of 24–27.9 is classified as overweight; a BMI ≥ 28.0 is defined as obesity. This research was approved by the Ethics Committee of the Capital Medical University (2014SY33). All subjects were voluntarily surveyed and provided signed informed consent.

### 2.2 Assessment of FAs intake

The good validity of food frequency questionnaire (FFQ) was found in the intake of dietary nutrients. Dietary intake of FAs was estimated by using a semiquantitative FFQ, which included 34 items (whole grain, red meat, pork, beef, mutton, chicken, fish, legume and legume product, milk, eggs, fruit and vegetables, nuts, sugared beverages, cooking oil, *etc*.), and the frequency (daily, weekly, monthly, yearly or never) and quantity of each consumption. The quantity of consumed food was estimated by using food models such as special charts and measuring rulers or cups. Trained interviewers helped all participants in completing the FFQ to ensure the accuracy of the collected data. Then all the data were collected with Epidata 3.1 by two investigators and consistency check were used to ensure the accuracy of the data. The all the data were converted to excel and the intake of total calories, protein, carbohydrate, fat, and FAs (including SFAs, MUFAs, PUFAs, and TFAs) per day was then calculated based on the China Food Composition Database (Yang, 2018).

### 2.3 Measurement of physical parameters

Body weight and height were measured with height and weight meter (Wuxi Weighing Apparatus Co., Ltd.). Waist and hip circumferences were measured at the umbilicus level and at the widest part of the hip, respectively, with a tape ruler. All data were rounded to the nearest millimeter.

### 2.4 Assessment of cognitive function

The MMSE and MoCA scales were wildly used to survey and evaluate the cognitive function of each subject in our previous study (An et al., 2018; Tao et al., 2019).MMSE scale is the most wildly used multi‐domain cognitive screening test containing 20 subtests (Folstein, Folstein, & McHugh, 1975). The total score of MMSE is 30 points, which is allocated to the five cognitive domains separately as following: 10 points for orientation, 6 points for memory, 5 points for computation, 6 points for language skills, 2 points for communication, 1 points for visual space ability. MocA scale was developed as the research increasing focused on prestage of dementia and the growing need for earlier diagnosis and management (Nasreddine et al., 2005). As another multi‐domain cognitive screening instrument, the total score of MoCA is also 30 points, which is allocated to the five cognitive domains separately as following: visual 5 points for space/execution ability, 3 points for naming, 6 points for attention, 3 points for language skills, 2 points for abstract thinking, 5 points for delayed recall and 6 points for orientation. The MMSE and MoCA questionnaires were carried out by trained physicians at community health service centers.

### 2.5 Plasma FAs profile determination

According to the method of our published study (Fan, Zhao, Ding, Xiao, & Ma, 2019), plasma FAs levels were measured by using fast gas chromatography (GC). Briefly, 100 μl of plasma sample was saponified by adding 100 μl eleven carbonate triglyceride and 1 ml KOH‐methanol solution; the mixed solution was then sealed with nitrogen and heated in a water bath at 60 ℃ for 10 min. After cooling down, the sample was esterified with boron trifluoride‐methanol reagent (at the same temperature for 40 min). Once the tubes were cooled, FA methyl esters were isolated by adding 1.5 ml of *n*‐hexane. 2 ml of saturated sodium chloride solution was then added. Finally, the tubes were centrifuged for 15 min at 3,000×g and dried with anhydrous sodium sulfate, and the clear *n*‐hexane top layer was transferred to an automatic injector vial. Fast GC analyses were then performed on a Shimadzu GC‐2010 Gas Chromatograph (Shimadzu). The identities of sample methyl ester peaks were determined by comparing their relative retention times with those of well‐known FA methyl esters standards. Quantification was performed by using standard normalization.

### 2.6 Statistical analysis

The Epidata 3.1 software was used for questionnaire input, and SPSS 23.0 was used for data cleaning and statistical analyses. One‐way ANOVA analysis followed by (LSD) least significant difference or Dunnett T3 was used to compare the waist circumference, hip circumference, waist‐to‐hip ratio, and BMI across different BMI groups. The *χ* ^2^ test for categorical variables was used to compare the demographic characteristics, smoking, and drinking status. Rank transformation followed by one‐way ANOVA analysis was used to compare the differences of dietary nutrients intake and plasma FA composition across different BMI groups. Spearman rank correlation analysis was used to investigate the correlation between dietary FA intake and plasma FA composition and cognitive function. For all analyses, *p* < .05 was considered to be statistically significant.

## 3 RESULTS

### 3.1 Characteristics of the study population

The general characteristics of all 672 subjects were collected with unified questionnaires. The study cohort included 305 males (45.39%) and 367 females (54.61%). In addition, 331 (49.3%) subjects held a diploma from secondary school and above, 522 participants were nonsmokers (77.7%), and 464 people did not drink alcohol (69.0%). The basic characteristics of all participants were summarized in Table 1. There was no significant difference in age and smoking status among normal‐weight, overweight and obese subjects (both *p* > .05). However, significant differences were found in gender, education, alcohol drinking status, and disease history among the three groups (both *p* < .05). Compared to the normal‐weight group, the total MoCA scores of the overweight and obese groups were both significantly lower (*p* < .05), while there was no significant difference in the MMSE total score across the three weight groups or for gender (*p* > .05).

**TABLE 1:** General characteristic of subjects in normal‐weight, overweight and obese groups

	Normal (*n* = 197)	Overweight (*n* = 260)	Obese (*n* = 215)	*p* Value
MMSE score	28.8 ± 1.5	28.6 ± 1.6	28.5 ± 1.8	.112
MoCA score	26.3 ± 2.3	25.7 ± 2.4*	25.7 ± 2.5*	.008
Gender (%)				.026
Male	24.3	42.6	33.1	
Female	33.5	35.4	31.1	
Age (year)	47.0 ± 7.3	49.2 ± 6.8*	48.9 ± 7.5*	.004
Educational level (%)				.001
Primary and below	18.0	52.0	30.0	
Junior high school	23.4	38.5	38.1	
Senior high school and above	36.3	36.9	26.9	
Smoking (%)				.075
Yes	22.0	41.3	36.7	
No	31.4	37.9	30.7	
Drinking (%)				.001
Yes	20.2	46.2*	33.7*	
No	33.4	35.3	31.3	
Hypertension (%)				<.001
Yes	16.8	45.2*	37.9*	
No	33.4	36.6	30.0	
Diabetes mellitus (%)				.016
Yes	22.9	30.0*	47.1*	
No	30.0	39.8	30.2	
Hyperlipidemia (%)				.002
Yes	13.2	44.0*	42.8*	
No	31.6	37.9	30.4	

Note

Descriptive statistics were performed to estimate the distribution of gender, age, education, smoking and drinking status, mean waist circumference, hip circumference, waist‐to‐hip ratio, and BMI in all subjects. Data for waist circumference, hip circumference, waist‐to‐hip ratio, and BMI represent the mean ± *SD* of each group. One‐way ANOVA followed by LSD or Dunnett T3 was used to compare age, MMSE score, MoCA score. *χ* ^2^ test for categorical variables was used to compare the demographic characteristics, smoking, drinking, hypertension, diabetes mellitus, and hyperlipidemia.

Abbreviations: MMSE, mini‐mental state examination; MoCA, montreal cognitive assessment.

* =*p* < .01 compared with normal subjects.

### 3.2 Assessment of dietary FAs intake

Table 2 showed the daily intake of macronutrients and FAs in the normal‐weight, overweight, and obese groups. The differences were not statistically significant in the intake of daily total calories, proteins, and fats among three groups. But Daily protein intake per kilogram of body weight varies significantly among three groups, which was lower in obesity group than that in normal (*p* < .05).Regarding to the intake of FAs, the obese group had a significantly higher intake of SFAs than the normal‐weight subjects (*p* < .05), but the intakes of TFAs, MUFAs, and PUFAs showed no significant difference among the normal‐weight, overweight and obese subjects.

**TABLE 2:** Daily intake of macronutrients and fatty acids in normal‐weight, overweight, and obese subjects

	Normal (*n* = 197)	Overweight (*n* = 260)	Obese (*n* = 215)	*p* Value
Total energy (kcal/day)	1,813.79 (1,289.43–2,435.56)	1,955.91 (1,427.87–2,563.16)	2,019.42 (1,464.98–2,695.75)	.058
Protein (g/day)	75.91 (51.45–107.25)	81.38 (61.03–110.36)	83.88 (59.55–114.02)	.087
Protein (g/kg BW/day)	1.26 (0.83–1.80)	1.18 (0.84–1.60)	1.00 (0.74–1.36)	.001
Fat (g/day)	49.66 (32.75–88.81)	56.59 (35.44–95.07)	60.50 (38.31–97.42)	.071
Carbohydrate (g/day)	246.35 (183.65–326.63)	255.06 (193.46–346.87)	256.64 (201.11–339.81)	0.304
Cholesterol (g/day)	345.00 (179.36–482.73)	372.17 (193.11–482.10)	369.42 (192.52–531.55)	.606
SFAs (mg/day)	13.01 (8.47–19.34)	14.80 (9.74–20.53)	15.77 (10.16–22.50)*	.041
MUFAs (mg/day)	16.94 (10.64–25.27)	18.83 (12.05–28.56)	19.63 (12.60–29.33)	.073
PUFAs (mg/day)	17.12 (9.65–29.36)	17.88 (11.35–31.38)	18.29 (11.00–30.24)	.475
TFAs (mg/day)	0.53 (0.25–0.79)	0.53 (0.27–0.78)	0.59 (0.32–0.90)	.108

Note

Data in the parentheses represented the interquartile range (IQR) of each factor. Rank transformation followed by One‐way ANOVA analysis was used to compare the differences across different BMI groups.

Abbreviations: BW, body weight;MUFAs, monounsaturated fatty acids; PUFAs, polyunsaturated fatty acids; SFAs, saturated fatty acids; TFAs, *trans* fatty acids.

* =*p* < .05, compared to normal‐weight subjects.

### 3.3 Plasma FAs profile

The plasma FA profiles of each BMI group were also investigated. Plasma FA profile was also different across BMI groups. The obese subjects had higher plasma levels of total SFAs, C14:0 and C16:0 compared to the normal‐weight participants (all *p* < .05, Table 3). The plasma level of C14:0, a type of SFA, was significantly up‐regulated in the overweight (*p* < .05; Table 3). The plasma levels of total MUFA, C16:1, and C18:1n9c in the overweight and obese group were significantly higher than that of the normal subjects (all *p* < .05; Table 3). Regarding the PUFA, however, the total plasma PUFA was not significantly different across all BMI groups (all *p* > .05); the C18:3n3 and *n*−3 PUFA were found higher in the obese group compared to both overweight and normal‐weight subjects (both *p* < .05; Table 3); the *n*−6/*n*−3 PUFAs in the obese subjects was lower than that in the normal subjects (*p* < .05; Table 3).

**TABLE 3:** Plasma fatty acids profiles of normal‐weight, overweight, and obese subjects

Unit (mg/ml)	Normal (*n* = 197)	Overweight (*n* = 260)	Obese (*n* = 215)	*p* Value
C14:0	0.016 (0.009–0.024)	0.017 (0.012–0.027)*	0.019 (0.013–0.028)**	.004
C16:0	0.64 (0.51–0.77)	0.67 (0.57–0.80)	0.70 (0.56–0.86)**	.021
C18:0	0.28 (0.23–0.32)	0.29 (0.24–0.33)	0.28 (0.24–0.34)	.314
Total SFAs	0.94 (0.77–1.11)	0.98 (0.83–1.15)	1.00 (0.83–1.24)*	.045
C16:1	0.030 (0.020–0.044)	0.032 (0.023–0.048)*	0.037 (0.023–0.058)**	.005
C18:1n9c	0.50 (0.39–0.67)	0.54 (0.43–0.70)*	0.57 (0.45–0.74)**	.007
Total MUFAs	0.56 (0.41–0.73)	0.58 (0.46–0.76)*	0.61 (0.48–0.82)**	.016
C18:2n6c	1.12 (0.93–1.31)	1.14 (0.97–1.38)	1.18 (0.98–1.43)	.105
C18:3n3	0.033 (0.015–0.079)	0.033 (0.014–0.098)	0.052 (0.020–0.121)**,^##^	.002
C20:3n6	0.030 (0.018–0.040)	0.034 (0.023–0.045)	0.033 (0.020–0.050)	.055
C20:4n6	0.25 (0.21–0.31)	0.26 (0.21–0.32)	0.24 (0.19–0.30)^##^	.021
C22:6n3	0.055 (0.043–0.074)	0.053 (0.039–0.070)	0.052 (0.040–0.074)	.449
Total PUFAs	1.54 (1.30–1.74)	1.57 (1.38–1.85)	1.56 (1.36–1.94)	.099
*n*−6 PUFAs	1.42 (1.20–1.63)	1.45 (1.25–1.70)	1.46 (1.21–1.78)	.198
*n*−3 PUFAs	0.106 (0.072–0.15)	0.105 (0.069–0.16)	0.126 (0.084–0.18)**,^##^	.004
*n*−6/*n*−3 PUFA	13.37 (8.99–19.03)	15.36 (9.27–20.58)	12.17 (8.25–18.04)^##^	.014

Note

Data in the parentheses represented the Interquartile range (IQR) of each FA. Rank transformation followed by One‐way ANOVA analysis was used to compare the differences across different BMI groups.

Abbreviations: MUFAs, monounsaturated fatty acids; PUFAs, polyunsaturated fatty acids; SFAs, saturated fatty acids.

* =*p* < .05, compared to normal‐weight subjects;

** =*p* < .01, compared to normal‐weight subjects.

## =*p* < .01, compared to overweight subjects.

### 3.4 Correlation between dietary FAs and cognitive function

We then investigated the potential correlations between dietary FAs intake and cognitive performance in normal‐weight, overweight and obese subjects in Table 4, respectively. We found that the intake of SFA, PUFA, and MUFA were negatively correlated with total MoCA score only in the normal‐weight group (all *p* < .05) but not in the overweight and obese groups (Table 4). The dietary intakes of SFA, PUFA, MUFA, and TFAs were not significantly associated with the MMSE score in all three groups.

**TABLE 4:** The correlation between dietary fatty acids profiles and cognitive performance in normal‐ weight, overweight and obese subjects (*n* = 672)

	SFAs		MUFAs		PUFAs		TFAs	
	*r*	*p* Value	*r*	*p* Value	*r*	*p* Value	*r*	*p* Value
Normal weight (*n* = 197)								
MMSE scale	−.091	.203	−.096	.179	−.114	.109	−.113	.113
MoCA scale	−.167*	.019	−.170*	.017	−.168*	.018	−.126	.078
Overweight (*n* = 260)								
MMSE scale	−.050	.426	−.035	.571	−.047	.453	−.017	.783
MoCA scale	−.041	.514	−.002	.972	.006	.922	−.066	.286
Obese (*n* = 215)								
MMSE scale	−.043	.527	−.040	.561	−.094	.172	−.117	.088
MoCA scale	−.006	.934	.029	.671	−.029	.667	−.065	.339

Note

Spearman rank correlation analysis was used to investigate the correlation between FAs intake and cognitive function.

Abbreviations: MMSE, mini‐mental state examination; MoCA, Montreal cognitive assessment scale; MUFAs, monounsaturated fatty acids; PUFAs, polyunsaturated fatty acids; SFAs, saturated fatty acids; TFAs, trans fatty acids.

* =*p* < .05.

### 3.5 Correlation between plasma FA profile and cognitive function

The correlations between the plasma levels of SFAs, MUFAs, PUFAs, n6/n3 PUFA ratio, and cognitive scores were summarized in Table 5. Interestingly, we found that the plasma SFA, MUFA, and PUFA were negatively correlated to both MMSE and MoCA scores in obese subjects (all *p* < .05, but these correlations were not established in normal‐weight and overweight subjects.

**TABLE 5:** The correlation between plasma fatty acids profiles and cognitive performance in normal‐ weight, overweight and obese subjects (*n* = 672)

	SFAs		MUFAs		PUFAs		n6/n3 PUFA ratio	
	*r*	*p* Value	*r*	*p* Value	*r*	*p* Value	*r*	*p* Value
Normal weight (*n* = 197)								
MMSE scale	−.115	.107	−.090	.211	−.121	.092	.046	.519
MoCA scale	.037	.603	.088	.217	.014	.845	.104	.145
Overweight (*n* = 260)								
MMSE scale	−.010	.867	.021	.742	.006	.922	.007	.906
MoCA scale	−.035	.569	−.009	.889	−.052	.401	.070	.258
Obese (*n* = 215)								
MMSE scale	−.206**	.002	−.155*	.023	−.241**	.000	.067	.331
MoCA scale	−.215**	.002	−.160*	.019	−.199**	.003	−.092	.178

Note

Spearman rank correlation analysis was used to investigate the correlation between FAs intake and cognitive function.

Abbreviations: MMSE, mini‐mental state examination; MoCA, Montreal cognitive assessment scale; MUFAs, monounsaturated fatty acids; PUFAs, polyunsaturated fatty acids; SFAs, saturated fatty acids.

* =*p* < .05,

** =*p* < .01.

## 4 DISCUSSION

Obesity is a significant public health problem and is associated with imbalanced diet intake (Wyckoff, Evans, Manasse, Butryn, & Forman, 2017). Imbalanced dietary FAs intake is an important factor for body fat accumulations in obese people. Accumulating evidence suggests an important role of healthy diets in maintaining normal brain functions (Eskelinen et al., 2008). The results of this study provided evidence that plasma SFAs, MUFAs, and PUFAs were associated with poorer cognitive functions in an obese population. These correlations suggested that variations in plasma FA composition might account for the declined cognitive function in obesity.

Excessive carbohydrates and FAs intake have been implicated in obesity (Okereke et al., 2012), which has been involved in the development of cognitive impairment (Naqvi, Harty, Mukamal, Stoddard, Vitolins & Dunn, 2011). FA plays a critical role in the structure and function of developing the nervous system. In this study we found the intake of SFA, PUFA, and MUFA were negatively correlated with total MoCA score in the normal‐weight group, but not in overweight and obesity group. Oleson et al., 2017 found that SFAs intake, in midlife, was correlated with poor memory performance (Oleson et al., 2017). It is expected that excess energy intake from dietary fat further alters free FA fluxes, reducing PUFAs and increasing SFAs in the circulation. Several reports suggest that SFAs activate TLR4‐dependent signaling pathways that increase inflammatory responses in microglia and induce brain inflammation, thus modulate cognitive impairments (Lancaster et al., 2018; Wang et al., 2012). These results suggested that dietary modification of fat intake might be an important target for preventing cognitive decline. Baym et al. investigated the cross‐sectional correlation of SFAs and omega‐3 (n23) with hippocampus‐dependent relational memory in prepubescent children, and they found that SFAs intake was negatively associated with both forms of memor, whereas omega‐3 FA intake was selectively positively associated with hippocampus‐dependent relational memory. Interestingly, excess dietary PUFAs and MUFAs were also related to lower cognitive performance in normal‐weight subjects in the present study. These results indicate the excess of FA intake contributed to poor cognitive performance no matter what type of FAs.

Many studies have suggested a close relationship between TFA intake and cognitive function. Devore et al. found that higher intake of TFA since midlife was highly associated with worse cognitive decline in women with type 2 diabetes (Devore et al., 2009). Morris *et al*. showed similar results that higher intake of TFA was linearly associated with greater decline in cognitive score over 6 years (Morris, Evans, Bienias, Tangney, & Wilson, 2004). However, we did not find the relationship between TFAs intake and cognitive performance in Chinese populations, which was not consistent with other researches. This may be due to the low intake of TFA in the Chinese population, which was is far lower than that in above population. Too low intake of TFA may not be enough to cause impairment of cognitive function.

In the present study, the plasma FA profile was tested in normal‐weight, overweight, and obese subjects by using GC. Since FA composition in either blood or erythrocytes can reflect dietary fat intake over approximately the past weeks and months (Devore et al., 2009; Morris et al., 2004), in particular, essential FAs (Guerendiain et al., 2018). But we did not analyze the relationship between dietary FA and plasma FAs (showed in Table S1). This may be due to plasma FAs are affected by many factors, such as disease, metabolic state, body composition, exercise, and other nutrient intake, et al. The results showed that the levels of total FFA, include SFAs and MUFAs, same as some certain FAs, like C16:0, C16:1, C18:1n9c, C18:3n3, and *n*−3 PUFAs. Peter Arner et al. found that obesity has a higher level of FFA (Arner & Rydénl, 2015), which is consistent with our study. Klein‐Platat et al. assumed that the reduction in plasma SFAs could have a beneficial effect on cognitive dysfunction. But they also found higher PUFAs were good for cognitive function (Klein‐Platat et al., 2005). Our results showed no difference of total PUFAs between normal‐weight and overweight and obesity subjects but increased *n*−3 PUFAs and MUFAs were found in obese subjects. We assume *n*−3 PUFAs and MUFAs content tended to increase and present protective effects in obesity. The altered FA levels observed in this study can also be explained by dysfunction of visceral adipose tissues, which found extreme visceral fat accumulation subject tend to have high SFA, MUFAs, *n*−6 PUFAs, and *n*−3 PUFAs. (Kang et al., 2017).

Regarding the associations between FA profile and cognitive performance, we found that plasma SFAs, PUFAs, and MUFAs were negatively correlated with poor cognition in obese subjects but not in normal‐weight and overweight populations. Analogously, Dullemeijer et al. also did not observe an association between n3 PUFA plasma concentration and memory and verbal fluency in healthy adults (Dullemeijer et al., 2007). Haapala et al. demonstrated that plasma PUFA is directly associated with cognition in overweight children but not in normal‐weight children, while PUFA may improve cognition of overweight children (Haapala et al., 2016). We speculated that the negative correlation between plasma PUFAs and cognitive function in the obese population may be attributed to an abnormal ratio of *n*−6 PUFA to *n*−3 PUFA. Another explanation for the direct associations of plasma MUFA with cognition in the present study may be that higher plasma MUFA level exhibited a protective effect on cognition. These data demonstrated that the effects of plasma FAs composition on the cognitive function in the obese population. The present study showed significant correlations between plasma SFA, PUFA and MUFA, and cognition based on MMSE and MoCA assessment in obese subjects. These data indicated that plasma FAs may contribute to the development of cognitive dysfunction caused by obesity.

Furthermore, in the present, significant differences were found in gender, education, and alcohol drinking status among normal‐weight, obesity, and obese groups, suggesting that male population tend to be overweight and obese more than female population. The overweight and obese subjects may intake more alcohol, indicating that excess alcohol intake can be a contributing factor to weight gain (Traversy & Chaput, 2015). We also found the overweight and obese subjects have lower education background than normal‐weight subjects.

### 4.1 Limitations

The information of dietary intakes of FAs was acquired with FFQs, which has several limitations, including a fixed list of foods, reliance on memory, and perception of portion sizes, may contribute to the inaccurate FAs intakes. In addition, measurement errors might be present when plasma FAs profile was measured by using GC, we attempted to correlate FFQ with plasma data and later with cognitive function, this could be the strongest limitation of the study. Furthermore, the confounding factors such as gender and education may also influence the FA intake.

## 5 CONCLUSION

From current data, we found higher plasma levels of SFA, PUFA, and MUFA in obese populations, which were associated with declined cognition. Lowering plasma FA levels may help maintaining normal cognitive functions in obese people.

## 6 ETHICS APPROVAL AND CONSENT

This research was approved by the Ethics Committee of the Capital Medical University (2014SY33). All subjects were voluntarily surveyed and provided signed informed consent.

## CONFLICTS OF INTEREST

The authors declare no conflict of interest.

## Supporting information

Table S1
