# Supplementary material for: Plasma fatty acid profile is related to cognitive function in obese Chinese populations (35–64 years): A cross‐sectional study
Source: Food Sci Nutr. 2020 Aug 7;8(9):4773–81. doi: 10.1002/fsn3.1738 (PMC7500792; doi:10.1002/fsn3.1738)
Supplement: Supplementary file 1 — Table S1 [file FSN3-8-4773-s001.docx]

Table S The correlation between dietary fatty acid intake and plasma fatty acids profiles in normal- weight, overweight and obese subjects (n=672)^1^

|  | Dietary SFAs | | Dietary MUFAs | | Dietary PUFAs | | Dietary TFAs | |
| --- | --- | --- | --- | --- | --- | --- | --- | --- |
| Plasma FAs | r | *p* | r | *p* | r | *p* | r | *p* |
| Normal weight (n=197) |  |  |  |  |  |  |  |  |
| SFAs | -0.089 | 0.215 | — | — | — | — | — | — |
| MUFAs | — | — | -0.067 | 0.348 | — | — | — | — |
| PUFAs | — | — | — | — | 0.031 | 0.662 | — | — |
| TFAs | — | — | — | — | — | — | -0.218 | 0.002* |
| Overweight (n=260) | — | — | — | — | — | — | — | — |
| SFAs | 0.030 | 0.635 | — | — | — | — | — | — |
| MUFAs |  |  | 0.055 | 0.374 | — | — | — | — |
| PUFAs | — | — | — | — | 0.087 | 0.163 | — | — |
| TFAs | — | — | — | — |  |  | -0.041 | 0.513 |
| Obese (n=215) | — | — | — | — | — | — | — | — |
| SFAs | 0.027 | 0.691 |  |  | — | — | — | — |
| MUFAs | — | — | 0.012 | 0.860 | — | — | — | — |
| PUFAs | — | — | — | — | 0.115 | 0.094 | — | — |
| TFAs | — | — | — | — | — | — | -0.099 | 0.147 |

^1^Spearman rank correlation analysis was used to investigate the correlation between dietary FAs intake and cognitive function. *=*P*<0.05. SFAs: saturated fatty acids; MUFAs: monounsaturated fatty acid; PUFAs: polyunsaturated fatty acid.
